# Physiological responses of Siberian sturgeon (*Acipenser baerii*) juveniles fed on full-fat insect-based diet in an aquaponic system

**DOI:** 10.1038/s41598-020-80379-x

**Published:** 2021-01-13

**Authors:** Matteo Zarantoniello, Basilio Randazzo, Valentina Nozzi, Cristina Truzzi, Elisabetta Giorgini, Gloriana Cardinaletti, Lorenzo Freddi, Stefano Ratti, Federico Girolametti, Andrea Osimani, Valentina Notarstefano, Vesna Milanović, Paola Riolo, Nunzio Isidoro, Francesca Tulli, Giorgia Gioacchini, Ike Olivotto

**Affiliations:** 1 Dipartimento di Scienze della Vita e dell’Ambiente, Università Politecnica delle Marche, via Brecce Bianche, 60131 Ancona, Italy; 2 Mj Energy srl Società Agricola, Contrada SS. Crocifisso, 22, 62010 Treia, MC Italy; 3 Dipartimento di Scienze Agro-Alimentari, Ambientali e Animali (Di4A), Università di Udine, via Sondrio 2/A, 33100 Udine, Italy; 4 Dipartimento di Scienze Agrarie, Alimentari ed Ambientali, Università Politecnica delle Marche, via Brecce Bianche, 60131 Ancona, Italy

**Keywords:** Metabolism, Reverse transcription polymerase chain reaction, Animal physiology

## Abstract

Over the last years, the potential use of Black Soldier Fly meal (BSF) as a new and sustainable aquafeed ingredient has been largely explored in several fish species. However, only fragmentary information is available about the use of BSF meal-based diets in sturgeon nutrition. In consideration of a circular economy concept and a more sustainable aquaculture development, the present research represents the first comprehensive multidisciplinary study on the physiological effects of a BSF diet during sturgeon culture in an aquaponic system. Siberian sturgeon (*Acipenser baerii*) juveniles were fed over a 60-days feeding trial on a control diet (Hi0) and a diet containing 50% of full-fat BSF meal respect to fish meal (Hi50). Physiological responses of fish were investigated using several analytical approaches, such as gas chromatography-mass spectrometry, histology, Fourier Transformed Infrared Spectroscopy (FTIR), microbiome sequencing and Real-time PCR. While aquaponic systems performed optimally during the trial, Hi50 group fish showed lower diet acceptance that resulted in growth and survival reduction, a decrease in hepatic lipids and glycogen content (FTIR), a higher hepatic *hsp70.1* gene expression and a worsening in gut histological morphometric parameters. The low feed acceptance showed by Hi50 group sturgeon highlighted the necessity to improve the palatability of BSF-based diet designed for sturgeon culture.

## Introduction

Siberian sturgeon (*Acipenser baerii*) is one of the most valuable species in aquaculture, due to the production of caviar and high-quality meat for human consumption. Restocking programs are also of great interest for this species since it is included in the list of endangered wild populations^1^. Compared to other sturgeon species, *Acipenser baerii* shows rapid growth rate, resistance to pathogens, a relatively short reproductive cycle (7–8 years) and can be reared using a wide range of diets and environmental conditions^2,3^. Owing to its features and high commercial value, intensive aquaculture of Siberian sturgeon is presently carried out in different parts of the world and, in recent years, several studies regarding farming-related biology^4^ and nutritional requirements^1,5^ have been performed. To ensure proper growth and constant productivity, aquafeeds commonly used in sturgeon’s rearing are mainly represented by high-energy diets largely based on fish meal (FM) and fish oil (FO) to meet proper protein and n3 highly unsaturated fatty acids requirement^6,7^. However, for a more sustainable aquaculture development, the use of FM and FO should be limited for both ecological and economic reasons^8^. For this reason, the discovery of novel nutritious and more sustainable ingredients for aquafeeds formulation is crucial^9^.

Over the last years, several ingredients have been evaluated as FM substitutes in sturgeon aquaculture, ranging from vegetable ones like soybean meal^5^, rice concentrate^10^, sesame oil cake, corn gluten^11^, and spirulina microalgae^12^ to animal ones such as poultry by-products^7^. Nowadays, with the goal of a further reduction of aquaculture’s environmental footprint, insect species like the Black Soldier Fly (*Hermetia illucens*; BSF) represent very promising candidates as FM alternatives^13^. The great interest in the BSF meal as aquafeed ingredient is due to their eco-friendly rearing in terms of land use, water consumption, CO_2_ emissions and on high feed conversion efficiency (BSF larvae are able to grow on low value organic by-products converting them into valuable biomass)^14,15^. Furthermore, insects like Diptera and Coleoptera are part of the natural diet of Siberian sturgeon^16^. It is well known that these insects possess bioactive compounds like chitin, which at certain concentrations are able to boost the fish immune system and promote gut microbiota diversification^17,18^. From the nutritional point of view, BSF larvae have a suitable protein content and the amino acid composition is similar to that of FM^19^. However, BSF meal fatty acid profile has also some disadvantages, such as a high content of saturated fatty acids (SFA) and an extremely low content in polyunsaturated (PUFA) ones^20^. PUFA are particularly important for fish since deficiencies in these compounds may cause a general deterioration of fish health, poor growth, low feed efficiency and often high mortality^21–23^. Previous studies demonstrated that a proper PUFA dietary content is essential to sustain both larval and adult Siberian sturgeon growth and welfare^24,25^; these compounds play a pivotal role in sturgeon’s fillet and caviar quality^26^.

Some recent studies tested different defatted BSF meal inclusion levels in aquafeed formulation for several fish species, but results on fish physiological responses are still controversial^27–30^. This topic, however, has scarcely been investigated in sturgeon aquaculture and most of the results are limited to zootechnical analyses^31–33^. Nowadays, several laboratory approaches (histology, molecular biology, gas chromatography and infra-red spectroscopy) are available to evaluate fish welfare and quality and represent valid tools to assess the inclusion of new ingredients, like insect meal, in aquafeed production^23,34–36^. In addition, the use of full-fat BSF meal is preferable to the highly defatted in order to reduce manufacturing costs^37,38^. This aspect has been recently addressed by Truzzi et al.^39^. These authors developed an enrichment procedure to increase insects’ PUFA content that allowed to include up to 50% of BSF prepupae meal compared to FM in zebrafish diet without impairing fish growth and welfare^38^. Because of this positive result, this same enriched full-fat BSF dietary inclusion percentage was chosen for the present study, expecting to obtain more promising results respect to Caimi et al.^32^ that evidenced a significant reduction of feed consumption and growth performance in Siberian sturgeon juveniles fed on a diet in which FM was 50% replaced with highly defatted BSF larvae meal.

In the present study, Siberian sturgeon juveniles were fed over a 60-days feeding trial on a control diet (based on FM and FO; Hi0) and a diet containing 50% of enriched BSF meal (according to Truzzi et al.)^39^ respect to FM (Hi50). Results obtained on zootechnical performances, fillet fatty acid composition, liver and gut integrity, expression of genes involved in fish growth, stress and immune response and gut microbiome represent the first multidisciplinary investigation on the physiological effects of BSF-based diets in sturgeon juveniles. Furthermore, this is the first feeding trial using insect-based diets performed in an aquaponics system. This green technology combines aquaculture (production of fish) with horticulture (vegetables production) saving energy, water and nutrients^40^, representing an important step for the development of a sustainable aquaculture in a future zero-waste generation^41,42^.

## Results

### Water chemistry

**Figure 1:**
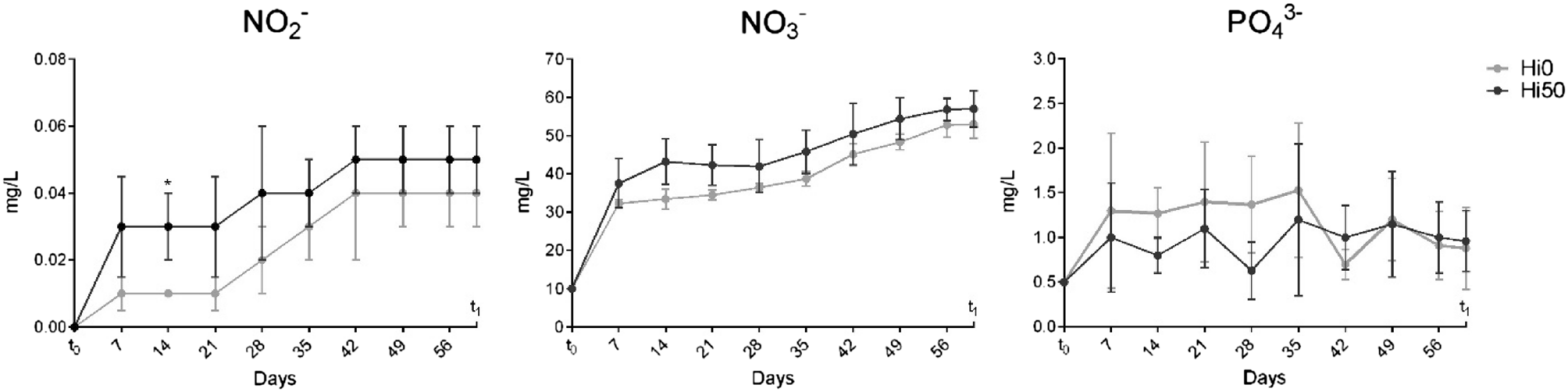
Trend of aquaponic system water parameters measured during the trial. Values are shown as mean (knots) ± SD (n = 3).

Nitrite (NO_2_^-^), nitrate (NO_3_^−^) and phosphate (PO_4_^3−^) weekly trends are shown in Fig. 1. No significant differences were detected between the two experimental groups. Ammonia values were lower than 0.05 mg/L for both Hi0 and Hi50 at each sampling time.

### Sturgeon survival and growth

Siberian sturgeon survival was significantly (p < 0.0001) lower in Hi50 (80 ± 4%) compared to Hi0 (97 ± 3%). Considering the specific growth rate (% weight gain day^−1^), Hi50 (1.8 ± 0.9%) was significantly (p < 0.0001) lower than Hi0 (2.9 ± 0.8%).

### Fatty acids composition

#### Diets

**Figure 2:**
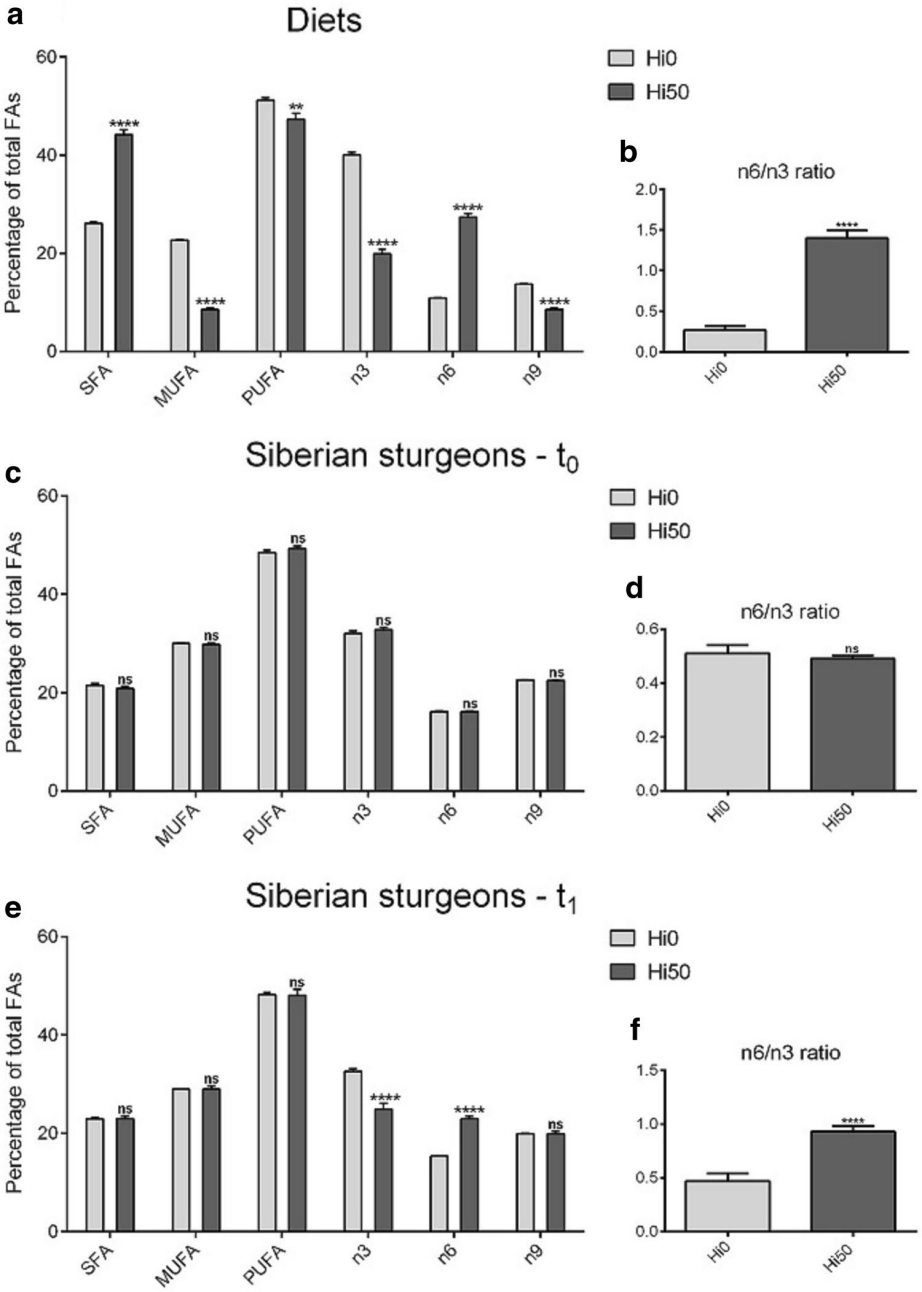
Percentage of SFA, MUFA and PUFA (as % of total FA) and omega 3 (n3), omega 6 (n6) and omega 9 (n9) FA contribution (%) to lipid profile**.** (**a**,**b**) Experimental diets including 0 and 50% of BSF meal respect to FM (Hi0 and Hi50); (**c**,**d**) Siberian sturgeon fillets at t_0_; (**e**,**f**) Fillets of Siberian sturgeon fed on the different diets at t_1_. Significant differences between Hi0 and Hi50, compared within the same FA class, are indicated as follows: *ns* non-significant; *p < 0.05; **p < 0.01; ***p < 0.001 and ****p < 0.0001. Values are shown as mean ± SD (n = 3 for experimental diets; n = 9 for sturgeon at both t_0_ and t_1_).

Figure 2a shows the percentages of fatty acids (FA) classes of the two experimental diets. The Hi50 diet resulted in a significantly (p < 0.0001) higher percentage of SFA (44.2 ± 1.0%) and significantly lower MUFA (8.6 ± 0.3%; p < 0.0001) and PUFA (47.3 ± 1.2; p < 0.01) compared to Hi0 diet (26.1 ± 0.3, 22.7 ± 0.1 and 51.2 ± 0.5% for SFA, MUFA and PUFA, respectively). In addition, the inclusion of BSF full-fat prepupae meal in the diet resulted in a significant (p < 0.0001) decrease of n3 (40.1 ± 0.5 and 19.9 ± 1.0% for Hi0 and Hi50, respectively) and n9 (13.7 ± 0.1 and 8.6 ± 0.3% for Hi0 and Hi50, respectively) percentages and a significant (p < 0.0001) increase in n6 percentage (10.9 ± 0.1 and 27.4 ± 0.7% for Hi0 and Hi50, respectively). Therefore, the n6/n3 ratio showed significant differences (p < 0.0001) between experimental diets (0.27 ± 0.05 and 1.40 ± 0.10 for Hi0 and Hi50, respectively; Fig. 2b).

Considering the specific FA composition (Table 1), the Hi50 diet was characterized by a significantly higher percentages of lauric (12:0), stearic (18:0), 7-hexadecenoic (16:1n9), linoleic (18:2n6) and α-linolenic (18:3n3) acids compared to the Hi0 diet. In the Hi0 diet palmitoleic (16:1n7), oleic (18:1n9), eicosapentaenoic (EPA, 20:5n3) and docosahexaenoic (DHA, 22:6n3) acids were significantly more abundant. Finally, the DHA/EPA ratio was significantly (p < 0.05) higher in the Hi50 diet compared to the Hi0 diet.

**Table 1:** Fatty acid composition (as % of total FA) of experimental diets and Siberian sturgeon at t_0_ and t_1_.

	Diets		Sturgeon			
	Hi0	Hi50	t_0_		t_1_	
			Hi0	Hi50	Hi0	Hi50
10:0	0.020 ± 0.001	0.570 ± 0.010****	0.005 ± 0.001	0.004 ± 0.001	0.003 ± 0.001	0.004 ± 0.001
12:0	0.18 ± 0.01	8.1 ± 0.7****	0.15 ± 0.04	0.12 ± 0.03	0.10 ± 0.03	2.35 ± 0.60****
13:0	0.06 ± 0.001	n.d	0.032 ± 0.001	0.029 ± 0.001	0.030 ± 0.002	0.044 ± 0.005
14:0	5.67 ± 0.06	4.6 ± 0.3**	2.80 ± 0.09	2.67 ± 0.04**	3.81 ± 0.12	3.73 ± 0.21^ns^
14:1n5	0.53 ± 0.01	n.d	0.34 ± 0.01	0.32 ± 0.01	0.41 ± 0.01	0.82 ± 0.07****
15:0	0.61 ± 0.01	0.58 ± 0.03 ns	0.61 ± 0.01	0.59 ± 0.01***	0.60 ± 0.01	0.57 ± 0.01****
15:1n5	n.d	n.d	n.d	n.d	n.d	n.d
16:0	14.5 ± 0.3	17.1 ± 0.6**	13.9 ± 0.1	13.7 ± 0.1***	14.7 ± 0.2	12.3 ± 0.1****
16:1n9	0.36 ± 0.01	6.3 ± 0.3****	0.49 ± 0.01	0.48 ± 0.01	0.49 ± 0.01	0.74 ± 0.06**
16:1n7	6.25 ± 0.04	n.d	4.37 ± 0.01	4.29 ± 0.01****	5.81 ± 0.11	5.60 ± 0.42^ns^
16:2n7	0.28 ± 0.01	n.d	0.30 ± 0.02	0.28 ± 0.01	0.13 ± 0.02	0.21 ± 0.01
17:0	0.53 ± 0.09	n.d	0.48 ± 0.09	0.55 ± 0.08	0.50 ± 0.01	0.41 ± 0.06
17:1n7	n.d	n.d	n.d	n.d	n.d	n.d
18:0	3.87 ± 0.01	13.1 ± 1.8***	3.21 ± 0.02	3.00 ± 0.02****	2.95 ± 0.39	3.44 ± 0.16**
18:1n9	9.94 ± 0.06	2.30 ± 0.20****	17.7 ± 0.1	17.6 ± 0.1*	16.5 ± 0.3	17.1 ± 0.2***
18:1n7	2.26 ± 0.01	n.d	2.66 ± 0.01	2.66 ± 0.02^ns^	2.91 ± 0.04	2.61 ± 0.17****
18:2n6	9.06 ± 0.03	27.4 ± 2.3***	12.9 ± 0.1	12.9 ± 0.1^ns^	12.5 ± 0.3	17.5 ± 1.3****
18:3n6	n.d	n.d	n.d	n.d	n.d	n.d
18:3n3	1.91 ± 0.06	3.4 ± 0.3**	2.51 ± 0.01	2.51 ± 0.01^ns^	2.03 ± 0.12	1.86 ± 0.06**
20:0	0.36 ± 0.01	n.d	0.24 ± 0.01	0.23 ± 0.01	0.19 ± 0.01	0.12 ± 0.02
20:1n9	1.83 ± 0.03	n.d	3.17 ± 0.04	3.19 ± 0.01^ns^	2.25 ± 0.12	1.80 ± 0.27***
20:2n6	0.33 ± 0.03	n.d	1.26 ± 0.05	1.24 ± 0.03^ns^	1.10 ± 0.02	1.93 ± 0.07****
20:3n6	0.26 ± 0.02	n.d	0.57 ± 0.08	0.53 ± 0.02 ns	0.53 ± 0.01	1.26 ± 0.07****
21:0	n.d	n.d	n.d	n.d	n.d	n.d
20:4n6	1.23 ± 0.01	n.d	1.48 ± 0.02	1.49 ± 0.02^ns^	1.27 ± 0.04	2.27 ± 0.16****
20:3n3	0.17 ± 0.01	n.d	0.43 ± 0.01	0.43 ± 0.01	0.30 ± 0.02	0.30 ± 0.04
20:5n3	17.3 ± 0.2	4.8 ± 0.4****	6.15 ± 0.04	6.26 ± 0.03****	9.29 ± 0.18	4.51 ± 0.05****
22:0	0.24 ± 0.01	n.d	0.081 ± 0.001	0.077 ± 0.003	0.063 ± 0.003	0.039 ± 0.009
22:1n9	1.53 ± 0.03	n.d	1.19 ± 0.05	1.19 ± 0.03^ns^	0.62 ± 0.06	0.25 ± 0.14****
23:0	n.d	n.d	n.d	n.d	n.d	n.d
24:0	n.d	n.d	n.d	n.d	n.d	n.d
22:6n3	20.7 ± 0.3	11.7 ± 1.5***	22.9 ± 0.1	23.6 ± 0.2****	21.0 ± 0.3	18.2 ± 1.7***
24:1n9	n.d	n.d	n.d	n.d	n.d	n.d
DHA/EPA	1.19 ± 0.03	2.5 ± 0.5*	3.7 ± 0.1	3.8 ± 0.1*	2.26 ± 0.06	4.0 ± 0.3****

Experimental diets included 0 and 50% of BSF meal respect to FM (Hi0 and Hi50). Siberian sturgeon fed diets including 0 and 50% of BSF meal (Hi0 and Hi50). Significant differences between Hi0 and Hi50 within rows and separately from diets, t_0_ and t_1_ are indicated as follows: *ns* non-significant; *p < 0.05; **p < 0.01; ***p < 0.001 and ****p < 0.0001. Values are shown as mean ± SD (n = 3 for experimental diets; n = 9 for sturgeon at both t_0_ and t_1_). Statistical analysis was performed only for FAs > 0.5% (FA with a percentage < 0.5% were excluded from any statistical analyses because their concentrations were close to the limit of detection).

#### Sturgeon

As shown in Fig. 2c,d, no significant differences were observed between the experimental groups at t_0_ in terms of either FA composition (SFA: 21.5 ± 0.4 and 20.9 ± 0.4%; MUFA: 30.0 ± 0.1 and 29.8 ± 0.2%; PUFA: 48.5 ± 0.5 and 49.3 ± 0.5%; n3: 32.0 ± 0.5 and 32.8 ± 0.5%; n6: 16.2 ± 0.1 and 16.2 ± 0.1%; n9: 22.6 ± 0.1 and 22.5 ± 0.1% for Hi0 and Hi50, respectively) or n6/n3 ratio (0.51 ± 0.03 and 0.49 ± 0.01 for Hi0 and Hi50, respectively). In terms of the specific composition, slightly significant differences were detected for some fatty acids due to physiological differences among fish (for specific details, see Table 1).

Considering FA content of Siberian sturgeon fillets at t_1_ (Fig. 2e), no significant differences were detected between the experimental groups in terms of SFA (22.9 ± 0.3 and 23.0 ± 0.5% for Hi0 and Hi50 respectively), MUFA (29.0 ± 0.1 and 29.0 ± 0.6% for Hi0 and Hi50, respectively), PUFA (48.2 ± 0.5 and 48.1 ± 1.2% for Hi0 and Hi50, respectively) and n9 (19.9 ± 0.1 and 19.9 ± 0.5% for Hi0 and Hi50, respectively) content. However, the Hi50 group was characterized by a significantly (p < 0.0001) lower n3 percentage (24.9 ± 1.2%) and a significantly (p < 0.0001) higher n6 percentage (23.0 ± 0.5%) than Hi0 (32.6 ± 0.5 and 15.4 ± 0.1% for n3 and n6, respectively). Consequently, the n6/n3 ratio (Fig. 2f) was significantly (p < 0.0001) higher in Hi50 (0.93 ± 0.05) compared to Hi0 (0.47 ± 0.07).

In terms of specific FA composition at t_1_ (Table 1), Hi50 showed significantly higher percentages of lauric (12:0), stearic (18:0), oleic (18:1n9), linoleic (18:2n6) and dihomo-γ-linolenic (20:3n6) acids than Hi0. Conversely, significantly higher percentages of α-linolenic (18:3n3), eicosapentaenoic (20:5n3) and docosahexaenoic (22:6n3) acids were detected in Hi0 respect to Hi50. The DHA/EPA ratio was significantly (p < 0.0001) higher in Hi50 compared to Hi0.

### Histology

Histological analyses at t_0_ were performed in order to evaluate liver and small intestine histological integrity at the beginning of the experiment. Sturgeons exhibited a homogeneous hepatic parenchyma with hepatocytes characterized by a moderate degree of intra-cytoplasmic lipid deposition (Supplementary Fig. S1a,b). The percentage of fat fraction (PFF) in the liver parenchyma did not show significant differences between Hi0 (49.2 ± 7.6%) and Hi50 (51.5 ± 9.4%) groups at t_0_. Histology of the small intestine (Supplementary Fig. S1c,d) evidenced a regular morphology of mucosal folds, with finger-shaped folds formed by a mono-stratified epithelial layer of enterocytes intercalated with goblet cells, followed by a thin submucosal layer surrounded by the outer muscular layer.

**Figure 3:**
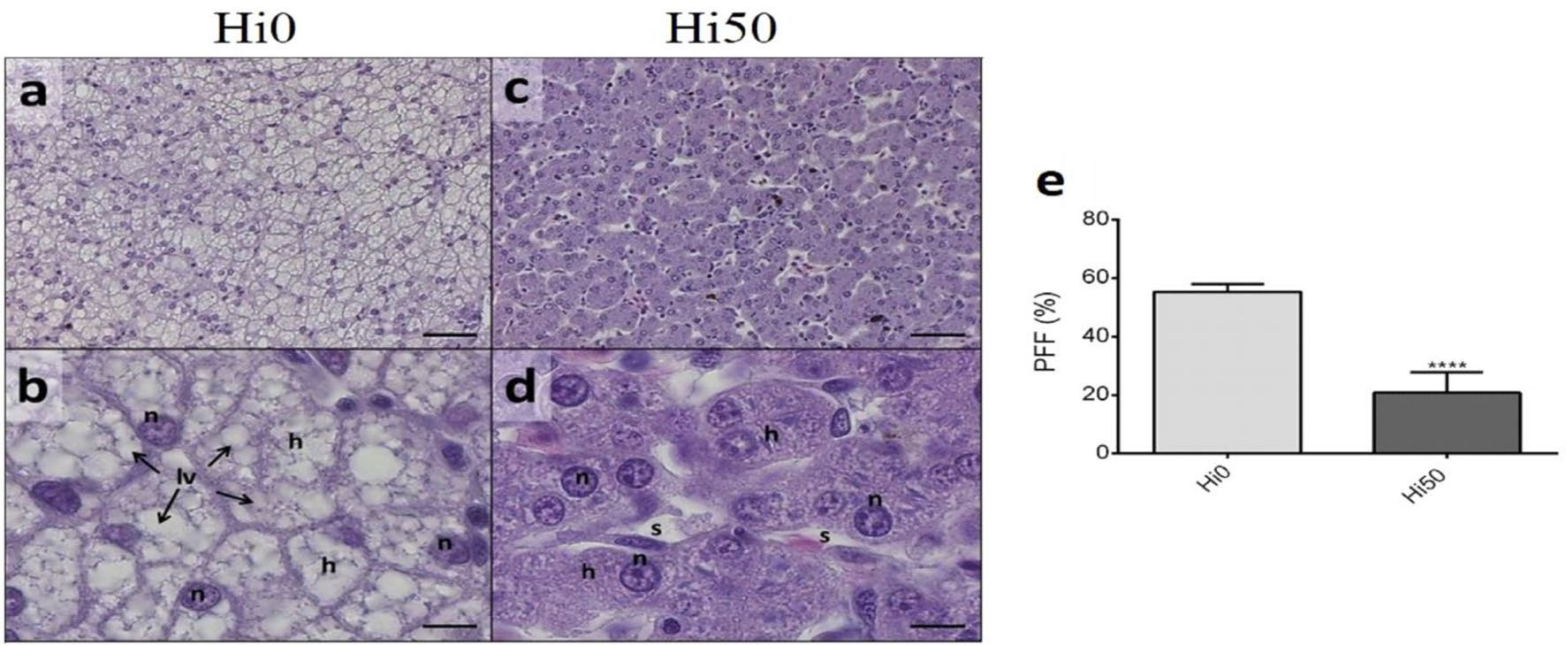
Example of liver histomorphology and percentage of fat fraction (PFF) in hepatic tissue of Siberian sturgeon at t_1_. (**a**,**b**) Hi0; (**c**,**d**) Hi50; (**e**) PFF. Histology scale bars: (**a**,**c**) 50 μm; (**b**,**d**) 10 μm. Letters: *h* hepatocyte, *n* nucleus, *lv* lipid vesicles, *s* hepatic sinusoids. For PFF, values are shown as percentage mean ± SD (n = 15). Significant differences between Hi0 and Hi50 are indicated as follows: ns, non-significant; *p < 0.05; **p < 0.01; ***p < 0.001 and ****p < 0.0001. Sturgeon fed diets including 0 and 50 of BSF meal (Hi0 and Hi50, respectively).

Liver analysis at t_1_ showed a considerable difference in parenchyma lipid accumulation between Hi0 and Hi50. Specifically, swollen hepatocytes filled of fat with limited cytoplasm were observed in livers of Hi0 sturgeons (Fig. 3a,b), while fat deposition was significantly reduced in the Hi50 group (Fig. 3c,d). PFF analysis at t_1_ confirmed this result highlighting significant differences in lipid deposition (p < 0.001) between Hi0 (55.3 ± 2.7%) and Hi50 (20.9 ± 7.0%) (Fig. 3e).

**Figure 4:**
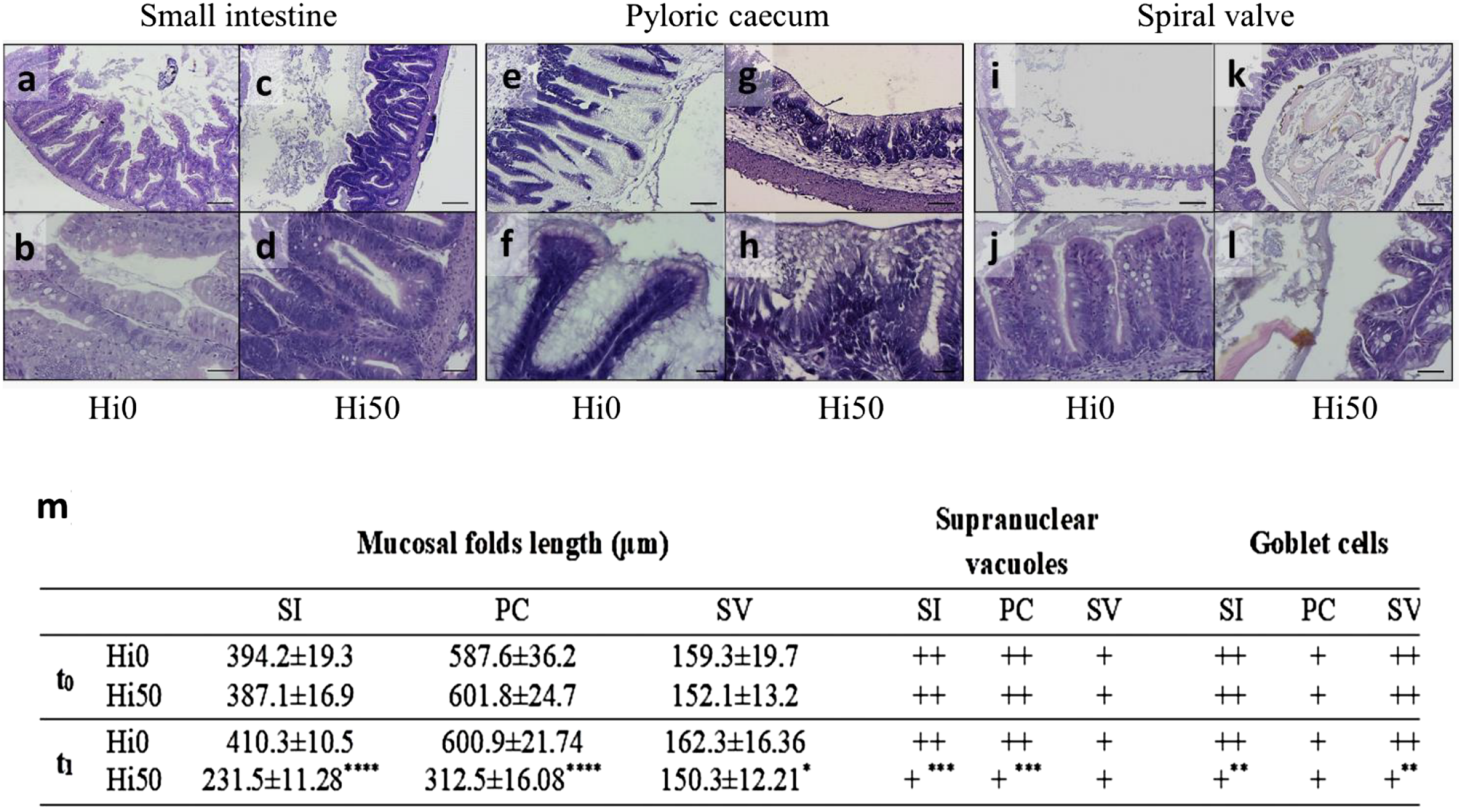
Siberian sturgeon’ small intestine (SI), pyloric caeca (PC) and spiral valve (SV) histology at t_1_ and histological indexes (mucosal folds length, supranuclear vacuoles abundance and goblet cells number) calculated in these gut tracts. (**a**–**d**) small intestine; (**e**–**h**) pyloric caecum; (**i**–**l**) spiral valve. Histology scale bars: (**a**,**c**,**e**,**g**) 100 μm; (**b**,**d**,**f**,**h**,**j**,**l**) 50 μm; (**i**,**k**) 200 μm. For histological indexes (**m**), values are showed as mean ± SD (n = 15). Scores: supranuclear vacuoles + scattered, ++ abundant; goblet cells + 0 to 4 per villus, ++  more than 4 per villus. Significant differences, calculated within the same sampling time, between Hi0 and Hi50 are indicated as follows: ns, non-significant; *p < 0.05; **p < 0.01; ***p < 0.001 and ****p < 0.0001. Sturgeon fed diets including 0 and 50 of BSF meal (Hi0 and Hi50, respectively).

**Figure 5:**
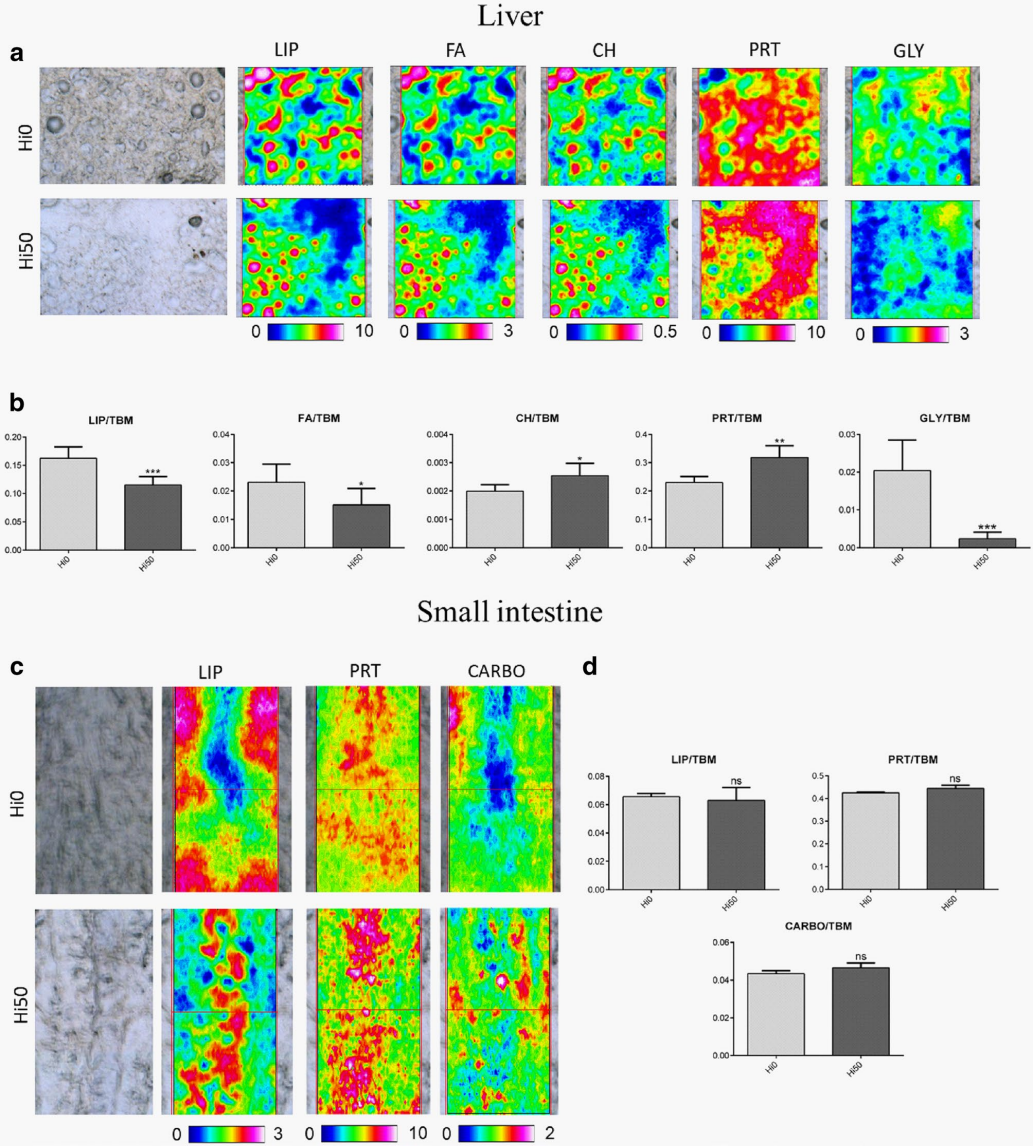
Spectral analysis of liver and small intestine samples of Siberian sturgeon collected at t_1_. (**a**) Microphotographs of liver samples and topographical distribution of: lipids (LIP maps), fatty acids (FA maps), unsaturated alkyl chains (CH maps), proteins (PRT maps), and glycogen (GLY maps) (map size 164 × 164 µm^2^). (**b**) Statistical analysis of liver biochemical composition: LIP/TBM (relative amount of total lipids), FA/TBM (relative amount of fatty acids), CH/TBM (degree of unsaturation in lipid alkyl chains), PRT/TBM (relative amount of total proteins) and GLY/TBM (relative amounts of glycogen). (**c**) Microphotographs of small intestine samples and topographical distribution of: lipids (LIP maps), proteins (PRT maps) and carbohydrates (CARBO maps) (map size 328 × 164 µm^2^). (**d**) Statistical analysis of small intestine biochemical composition: LIP/TBM (relative amount of total lipids), PRT/TBM (relative amount of total proteins) and CARBO/TBM (relative amount of carbohydrates). Significant differences between Hi0 and Hi50 are indicated as follows: *ns* non-significant; *p < 0.05; **p < 0.01; ***p < 0.001 and ****p < 0.0001. Data are reported as mean ± SD (n = 6). Sturgeons fed diets including 0 and 50 of BSF meal (Hi0 and Hi50, respectively).

Representative histological images of small intestine (SI), pyloric caecum (PC) and spiral valve (SV) are shown in Fig. 4. In the morphometric analysis of these gut tracts (Fig. 4m), no significant differences were detected between the groups at t_0_, while mucosal folds atrophy, with a significant reduction of folds length, was observed at t_1_ in Hi50 SI (Fig. 5c,d; p < 0.0001), PC (Fig. 4g,h, p < 0.0001) and SV (Fig. 4k,l, p < 0.05) compared to the Hi0 group (Fig. 4a,b,e,f,i,j for SI, PC and SV, respectively). In addition, a significant (p < 0.0001) reduction of supranuclear vacuoles in SI and PC and a significant (p < 0.01) reduction in the relative abundance of goblet cells in SI and SV were observed in Hi50 compared to Hi0.

### FTIR analysis

The spectral analysis of liver samples collected from Siberian sturgeons at t_1_ showed differences in the biochemical composition between Hi0 and Hi50. The IR maps (Fig. 5a), as well as the statistical analysis of specific band area ratios (Fig. 5b), showed a significant decrease of total lipids (LIP maps; LIP/TBM, p < 0.001), fatty acids (FA maps; FA/TBM, p < 0.05) and glycogen (GLY maps; GLY/TBM, p < 0.01) in Hi50 compared to Hi0. An increase in unsaturated lipids (CH maps; CH/TBM, p < 0.05) and proteins (PRT maps; PRT/TBM, p < 0.01) in Hi50 was also observed.

For the small intestine samples, both the IR maps (LIP, PRT and CARBO maps; Fig. 5c) and the statistical analysis of specific band area ratios (LIP/TBM, PRT/TBM and CARBO/TBM; Fig. 5d) did not show significant modifications between Hi0 and Hi50.

### Sturgeon gut microbiome

**Figure 6:**
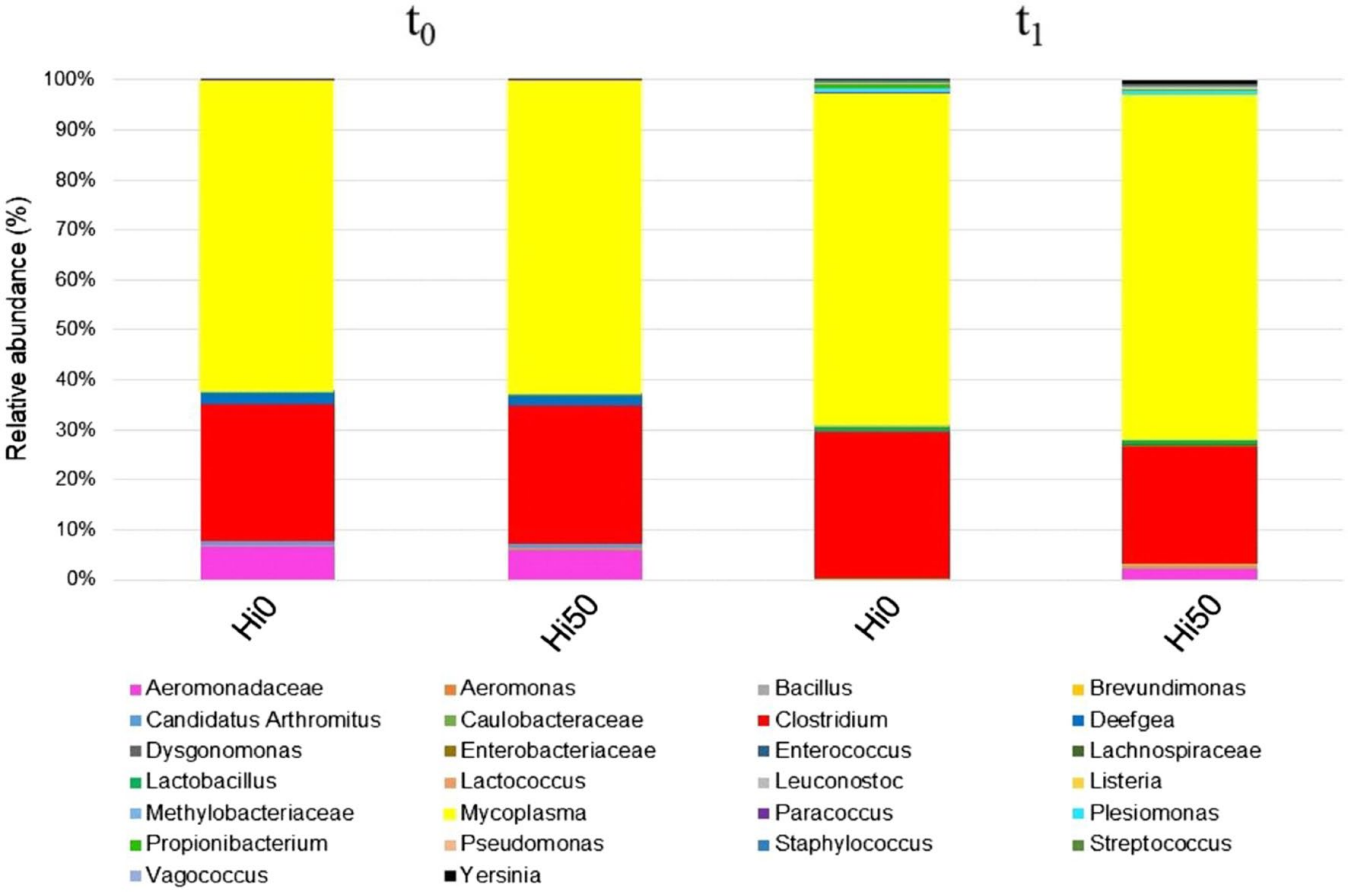
Relative abundances (%) of bacterial community in Siberian sturgeon gut samples at both t_0_ and t_1_ as identified by MiSeq Illumina. Sturgeon fed diets including 0 and 50 of BSF meal (Hi0 and Hi50, respectively).

No significant differences were observed in the alpha diversity values (Shannon, Chao1 and number of OTUs) between Hi0 and Hi50 at both t_0_ and t_1_. However, a higher number of bacterial groups at genus or family level were identified in Hi50 (22 groups) compared to Hi0 (17 groups) samples at t_1_. Relative abundances of bacterial taxa were examined to determine the effect of the diet on gut microbiota composition. The average values (Fig. 6) of the biological replicates at both sampling times were found to be very similar. The taxonomic analysis showed the dominance (> 58%) of *Mycoplasma* in all samples analysed, followed by *Clostridium*, with relative abundances comprised between 22.64% (Hi50, t_1_) and 28.27% (Hi0, t_1_). Aeromonadacean bacteria were found in gut samples from both Hi0 and Hi50 at t_0_ (about 6%), and exclusively in Hi50 sampled at t_1_ with the relative abundance of 2.08%. Bacteria of the genus *Deefgea* were present in both experimental groups exclusively at t_0_ with relative abundance of about 2%. Additional bacteria were detected sporadically in some samples, with a relative abundance < 1%. *Lactobacillus*, *Paracoccus*, *Propionibacterium* and *Streptococcus* were identified solely at t_1_ in both experimental groups, while *Listeria* was found only in Hi50.

### Real-time PCR results

Real-time PCR analyses were performed on liver samples in order to test the expression of genes involved in fish growth (*igf1*) and stress response (*hsp70.1*). Gene expression of *tnfa* was investigated in intestine samples.

**Figure 7:**
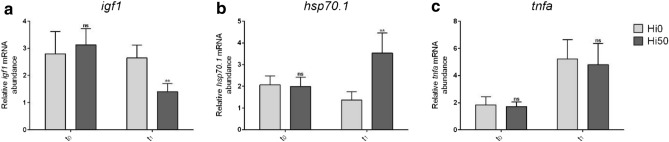
Relative mRNA abundance of genes analysed at t_0_ and t_1_ in Siberian sturgeon. (**a**) *igf1* and (**b**) *hsp70.1* were analysed in liver samples; (**c**) *tnfa* was analysed in intestine samples. Fish fed diets including 0 and 50 of BSF meal (Hi0 and Hi50, respectively). Significant differences between Hi0 and Hi50, compared within the same sampling time, are indicated as follows: *ns* non-significant; *p < 0.05; **p < 0.01; ***p < 0.001 and ****p < 0.0001. Values are shown as mean ± SD (n = 5).

As shown in Fig. 7, the expression of the genes analysed did not show significant differences between the experimental groups at t_0_. At t_1_, results evidenced a significant (p < 0.01) downregulation for *igf1* (Fig. 7a) and a significant (p > 0.01) upregulation for *hsp70.1* (Fig. 7b) in Hi50 compared to Hi0. For *tnfa* (Fig. 7c), no significant differences in gene expression were detected between the experimental groups.

## Discussion

The inclusion of BSF meal in aquafeed, as well as the physiological responses of fish, have been recently investigated in several important commercial species like Atlantic salmon (*Salmo salar*), rainbow trout (*Oncorhinchus mykiss*) and European seabass (*Dicentrarchus labrax*)^28,30,43^.

Information available in this field for the Siberian sturgeon is still fragmentary, in fact completely lacking if related to aquaponic systems. Previous studies, based on a limited number of laboratory approaches, highlighted that a defatted BSF prepupae meal dietary inclusion level higher than 25% impaired fish growth and welfare^31–33^. Based on these results and previous studies which demonstrated the possibility to use higher full-fat dietary BSF meal inclusion levels^23,38^ after an enrichment procedure of the insect biomass^39^, the present study aimed to: (i) test, for the first time, a 50% enriched full-fat BSF prepupae meal inclusion level in a practical diet for juvenile sturgeons in an aquaponic system; (ii) apply a set of laboratory techniques to examine comprehensively the physiological responses of the fish.

The results showed that the inclusion of a 50% enriched full-fat BSF prepupae meal negatively affects fish growth and survival. These conclusions are supported by the expression of the growth markers analysed and agree with previous studies reporting that inclusion of BSF meal levels higher than 40% often impairs fish growth and welfare^32,36^. A possible explanation of these differences between the two experimental groups can be related to the FA composition of the diets. Şener et al.^44^ reported high EPA and DHA levels in Russian sturgeon fed on diets rich in linoleic acid. In agreement with this study, our FA analyses showed the ability of sturgeon to convert linoleic acid (18:2n6) and α-linolenic acid (18:3n3) to EPA and DHA by desaturation and elongation enzymatic pathways^45,46^. Since biochemical conversions require expenditure of energy by the fish, they can explain the observed growth delay in Hi50 compared to Hi0^47^.

It should be pointed out, however, that a lower diet acceptance was observed in the Hi50 group compared to Hi0 and the laboratory analysis performed suggest that the fish entered a fasting condition. Fasting is normally characterized by a growth reduction due mainly to a decrease in IGFs production by hepatocytes^48^. In turn, these changes induce hepatic lipolysis to provide peripheral tissue with free FA as energy source^48,49^. A similar *scenario* was observed in the present study: Hi50 growth reduction was coupled with a lower *igf1* gene expression and a scarce lipid accumulation in the hepatic parenchyma. These results are also supported by PFF calculation and FTIR analysis which showed an overall decrease in both total lipids (LIP/TBM) and fatty acids (FA/TBM) in the Hi50 liver samples compared to Hi0. Furthermore, a severe reduction of hepatic glycogen, which represents the first energy reserve mobilized to face food restrictions^50,51^, was detected by FTIR in Hi50 compared to Hi0. Furné et al.^52^ demonstrated that the Adriatic sturgeon (*Acipenser naccarii*) responded to fasting with a precocious mobilization of hepatic glycogen and a high hepatic lipid-degradation capacity. Accordingly, our results represent a strong evidence that fish entered a fasting period. In addition, since malnutrition or fasting are nowadays considered stressors^22,53,54^, this stressful situation is fully supported by the higher hepatic *hsp70.1* gene expression detected in Hi50 compared to Hi0.

Histological analyses of intestinal tracts are also useful to provide evidence of fasting *status* in fish. Fasting is usually associated to a reduction in mucosal fold number and height, reduction in supra-nuclear lipid droplets and reduction in goblet cells numbers^55,56^. Accordingly, our Hi50 group showed atrophy of mucosal folds and a dramatic decrease of enterocyte vacuolization and goblet cells number compared to Hi0. In a study performed by Caimi et al.^33^ on Siberian sturgeon over a 118-days period, lower (37.5%) levels of defatted BSF meal dietary inclusion did not show these negative effects on spiral valve and liver histology. On the other hand, in a 60-days feeding trial, a 15% dietary inclusion of full-fat BSF prepupae caused a thinning of the mucosa and a parallel thickening of the muscular layer in the proximal intestine while not affecting villus height^31^. Overall, our analyses of the intestine histology and *tnfa* gene expression did not reveal any sign of inflammation in either of the experimental groups. Similar results were reported in other studies^30,36,57^ that evidenced a positive role of BSF meal dietary inclusion on fish gut welfare. BSF meal contains lauric acid and chitin, that possess anti-inflammatory and immune-boosting properties^58^. BSF meal is also known to rise biodiversity in the fish microbiome, necessary to improve fish health, metabolism, nutrition and immunity^17,59^. Our Hi50 diet contained higher percentages of lauric acid and chitin and supported a higher number of bacterial groups (at genus or family level) in the fish gut compared to Hi0, possibly explaining the absence of intestinal inflammatory events. The dominance of *Mycoplasma,* followed by *Clostridium*, was observed in all samples analysed, regardless of the diet. An increased relative abundance of *Mycoplasma* was detected by Rimoldi et al.^59^ in the autochthonous gut microbiota of rainbow trout fed with BSF-based diets. This author attributed the beneficial action on host health to the production of antibacterial compounds, such as lactic and acetic acid.

In conclusion, the present study demonstrated the feasibility of aquaponic systems for sturgeon culture and for testing new aquafeed ingredients like insect meal. However, the general low feed acceptance showed by the Hi50 group fish compared to Hi0 indicates the need of further studies aimed at improving the palatability of BSF-based diets intended for sturgeon culture.

## Methods

All methods were carried out in accordance with relevant guidelines and regulations.

### Ethics

All zootechnical trials were conducted in agreement with the Italian legislation on experimental animals and were approved by the Ethics Committee of the Università Politecnica delle Marche (Aut. No. 01/2019).

### Insects rearing and fish diet production

For details on insects rearing and fish diet production, see Supplementary Information section.

### Fish, aquaponic system and experimental design

The 60-days feeding trial was conducted at the aquaponics facility “Cooperativa Agricola Tanto Sole” (Treia, Macerata, Italy). Juvenile Siberian sturgeons*,* purchased from Azienda Agricola Pisani Dossi s.s., Cisliano, MI, Italy), were acclimated for 1 week in a single 500 L tank equipped with mechanical, biological and UV filtration (Panaque, Viterbo, Italy). Tank temperature was 18 ± 0.5 °C; ammonia (NH_3_) and nitrite (NO_2_^−^) were < 0.05 mg/L and nitrate (NO_3_^−^) 10 mg/L, according to sturgeon rearing requirements^60^. At the end of the acclimation period, fish were randomly allocated into six Media Based Aquaponic Systems (80 specimens per tank). Each aquaponic system consisted of a 1.56 m^2^ hydroponic unit for plants cultivation and a 600 L fish tank, for a total volume of 720 L of water.

#### Fish unit

The six systems were maintained at constant temperature (18.0 ± 0.5 °C) by chillers TK500 (Teco, Ravenna, Italy). Evaporated water was replaced on request and the systems were subjected to a natural photoperiod (11L/13D). Water samples were collected weekly in order to test ammonia (NH_3_), nitrite (NO_2_^−^), nitrate (NO_3_^−^) and phosphate (PO_4_^3−^) using Hanna reagents and a HI83399 spectrophotometer (Hanna instruments, Villafranca Padovana, Italy).

#### Hydroponic unit

Each hydroponic unit was filled with expanded clay with biological and mechanical filtration function^40^, necessary to guarantee a physical support for plant growth. Specifically, in each hydroponic unit, 16 lettuce (*Lactuca sativa*; initial weight: 2.95 ± 0.5 g) and 3 celery (*Apium graveolens*; initial weight: 20.8 ± 5.0 g) seedlings were planted two days before introduction of the fish (density = 12 plants/m^2^). Recirculating water flow from the fish tank to the hydroponic unit was regulated by a 1900 L/h pump (Eheim GmbH & Co, Deizisau, Germany) completing 3 water renewals per hour. Specifically, water was pumped from the fish tank to the hydroponic unit, and then returned to the fish unit through a siphon. The siphon was equipped with further synthetic foam for extra mechanical filtration (foam was cleaned once per week).

#### Feeding trial

At the beginning of the experiment (t_0_), the six aquaponic systems were randomly assigned to the experimental groups (Hi0, Hi50) according to an experimental design with triplicate tanks per dietary treatment. Feeding trial duration was 60 days, in which sturgeons almost triplicated their weight and were fed as follows: fish fed on the 0% of BSF meal diet (Hi0 group); fish fed on the diet including 50% of BSF full-fat prepupae meal (Hi50 group). Feed particle were 0.5–1 mm in size. Sturgeons were fed three times a day the experimental diets (3% body weight daily). At the beginning (t_0_) and at the end of the feeding trial (t_1_), after a 10-h fasting period, the required fish were sampled, euthanized with a lethal dose of MS222 (0.3 g/L; Merck KGaA, Darmstadt, Germany) and properly stored for further analyses.

### Biometry

For growth measurements, 60 sturgeons per dietary group (n = 3) at both t_0_ and t_1_ were randomly collected from the different tanks. Wet weight was measured with an OHAUS Explorer (OHAUS Europe GmbH, Greifensee, Switzerland) analytical balance (precision 0.1 mg). The specific growth rate (SGR) was calculated as follows: SGR% = [(lnW*f* – lnW*i*)/t) × 100, where W*f* is the wet weight determined at t_1_, W*i*, the wet weight determined at t_0_, and t, the number of days (60). During the trial, dead fish were removed and recorded to estimate the final survival rate.

### Fatty acid composition

Lipid content and fatty acid composition of experimental diets (n = 3) and fish fillet (9 sturgeon per dietary group; n = 3) were determined after sample homogenization (homogenizer MZ 4110, DCG, Eltronic, Monza, Italy) and freeze-drying (Edwards EF4, Crawley, Sussex, England). Lipid extraction was carried out on lyophilized powders with the Folch method (1957)^61^ for diets and with Microwave-Assisted Extraction (MAE) method for fish^62^. All lipid extracts were evaporated under laminar flow inert gas (N_2_) until constant weight to determine lipid content; then, they were re-suspended in 0.5 mL of n-epthane for fatty acid analysis. Fatty acid methyl esters (FAMEs) were prepared according to Canonico et al.^63^ using methyl ester of nonadecanoic acid (19:0; Dr. Ehrenstorfer GmbH, Augsburg, Germany) as internal standard. A gas-chromatographic (GC) system (Agilent-6890, Milano, Italy) coupled with a Mass Selective Detector (MS) (Agilent-5973N quadrupole, Milano, Italy) was used to determine FAMEs. A CPS ANALITICA CC-wax-MS (30 m × 0.25 mm ID, 0.25 μm film thickness) capillary column was used to separate FAMEs. Instrumental conditions were set up according to Truzzi et al.^64^. Analyses were carried out on three aliquots per sample. For each aliquot, at least three runs were performed on the GC–MS.

### Histology

Liver, small intestine and spiral valve from 15 different sturgeons per dietary group (n = 3) were randomly collected at both t_0_ and t_1_ and processed according to Piccinetti et al.^65^. For details, see Supplementary Information section. In order to evaluate the percentage of fat fraction in the liver (PFF), three sections per fish (15 fish per dietary group; n = 3), at 100 µm intervals, were acquired and analysed by mean of the ImageJ software setting an homogeneous threshold value according to Zarantoniello et al.^38^. Non evaluable areas on sections, such as blood vessels and bile ducts, were not considered. Results were reported as mean ± SD of the area occupied by fat on the total hepatic parenchyma analysed on the section. A semi-quantitative evaluation was performed on small intestine, pyloric caecum and spiral valve morphology based on mucosal folds height, supranuclear vacuolization of enterocytes and abundance of goblet cells as previously described in Urán et al.^66^. Specifically, for the morphometric evaluation of mucosal folds height, ten transversal sections per fish (15 fish per dietary group) of small intestine, pyloric caecum and spiral valve, at 200 μm intervals, were analysed as described in Cardinaletti et al.^28^. All the undamaged and non-oblique folds were measured (at least 150 measurements per fish) using ZEN 2.3 software (Carl Zeiss Microscopy GmbH) and measurements were reported as height mean ± SD (µm)^28^. For the semi-quantitative analysis of supranuclear vacuoles and goblet cells, 3 whole intestine circular transversal sections per fish (15 fish per dietary group), at 200 μm intervals, were analysed. The sections were analysed by experienced staff in two independent blinded evaluations and an arbitrary unit was assigned as described in Panettieri et al.^67^. Scores were assigned as follows: supranuclear vacuoles +  = scattered, +  +  = abundant; goblet cells +  = 0/4 per villus, +  +  > 4 per villus.

### FTIR measurements

Samples of liver and small intestine collected at t_1_ from 6 different sturgeons per dietary group (n = 3), were quickly dissected and immediately frozen at − 80 °C. Samples were then prepared for infrared spectroscopy (IR) measurements^68^ as reported in Supplementary Information section.

### Sturgeon gut microbiome

#### RNA extraction and cDNA synthesis

Gut samples from Hi0 and Hi50 groups were collected at t_0_ and t_1_. Specifically, 9 different sturgeons per dietary group (n = 3) were collected and processed as previously described by Zarantoniello et al.^38^. The obtained cell pellets were covered with RNA later Stabilization Solution (Ambion, Foster City, CA, USA) and stored at − 80 °C until the extraction of total microbial RNA performed by Quick-RNA Miniprep kit (Zymo Research, CA, USA). The quantity and purity of the extracted RNA were checked using a Nanodrop ND 1000 (Thermo Fisher Scientific). Moreover, the absence of residual DNA contamination was checked by PCR as described by Garofalo et al.^69^. Each sample RNA (10 μL) was reverse-transcribed in cDNA using oligo (dT) and random hexamer primers from SensiFAST cDNA Synthesis Kit for RT-qPCR (Bioline, London, UK).

#### 16S rRNA gene amplicon target sequencing

The portion of 16S rRNA gene (V3–V4 region) from each sample cDNA was amplified by PCR as previously described by Klindworth et al.^70^. The PCR products were further processed and sequenced by MiSeq Illumina instrument (Illumina, San Diego, California, USA) following the procedure detailed by Osimani et al.^18^.

### Molecular analyses

#### RNA extraction, cDNA synthesis and real-time PCR

Total RNA extractions from liver and small intestine samples from 15 sturgeons per dietary group (n = 3) at both t_0_ and t_1_ were performed using RNAzol RT reagent (R4533, Merck KGaA) according to Olivotto et al.^71^ and Vargas-Abùndez et al.^72^. For details on methods and primers sequences (reported in Supplementary Table S2), see Supplementary Information section.

### Statistical analysis

All data (except for microbiome) were analysed by t-test, with diet as the explanatory variable and presented as mean ± SD. The statistical software package Prism5 (GraphPad Software) was used. Significant differences between Hi0 and Hi50 were indicated as follows: ns, non-significant; *p < 0.05; **p < 0.01; ***p < 0.001 and ****p < 0.0001. For microbiome bioinformatics analyses, raw reads were first merged with the FLASH software and analysed with the QIIME 1.9.0 software^73^; the detailed pipeline was described by Ferrocino et al.^74^. OTUs clustering was obtained at 97% of similarity and centroids sequencing were mapped against the Greengenes 16S rRNA gene database. OTU tables generated by QIIME were rarefied at the lowest number of reads and showed the highest reached taxonomic resolution. The vegan package of R was used for the alpha diversity calculation.

## Supplementary Information

Supplementary Information.
