# Supplementary material for: Physiological responses of Siberian sturgeon (Acipenser baerii) juveniles fed on full-fat insect-based diet in an aquaponic system
Source: Sci Rep. 2021 Jan 13;11:1057. doi: 10.1038/s41598-020-80379-x (PMC7806854; doi:10.1038/s41598-020-80379-x)
Supplement: Supplementary file 1 — Supplementary Information. [file 41598_2020_80379_MOESM1_ESM.docx]

**SUPPLEMENTARY INFORMATION**

**Physiological responses of Siberian sturgeon (*Acipenser baerii*) juveniles fed on full-fat insect-based diet in an aquaponic system**

Matteo Zarantoniello^1^, Basilio Randazzo^1^, Valentina Nozzi^2^, Cristina Truzzi^1^, Elisabetta Giorgini^1^, Gloriana Cardinaletti^3^, Lorenzo Freddi^1^, Stefano Ratti^1^, Federico Girolametti^1^, Andrea Osimani^4^, Valentina Notarstefano^1^, Vesna Milanović^4^, Paola Riolo^4^, Nunzio Isidoro^4^, Francesca Tulli^3^, Giorgia Gioacchini^1^ & Ike Olivotto^1*^

^1^ Dipartimento di Scienze della Vita e dell’Ambiente, Università Politecnica delle Marche, via Brecce Bianche, 60131Ancona, Italy.

^2^ Mj Energy srl Società Agricola, Contrada SS. Crocifisso, 22, 62010 Treia (MC), Italy.

^3^ Dipartimento di Scienze Agro-Alimentari, Ambientali e Animali (Di4A), Università di Udine, via Sondrio 2/A, 33100 Udine, Italy.

^4^ Dipartimento di Scienze Agrarie, Alimentari ed Ambientali, Università Politecnica delle Marche, via Brecce Bianche, 60131 Ancona, Italy.

* i.olivotto@univpm.it

**Insects rearing**

Insects were reared on coffee by-product (coffee silverskin) obtained from the roasting process at Saccaria Caffè SRL, Marina di Montemarciano, Italy. Coffee silverskin was processed as already described by Zarantoniello et al. (2020)^38^ and the substrate was enriched with a 10% (w/w) of *Schizochytrium* sp^39^.

Six days old larvae [purchased from Smart Bugs s.s. Ponzano Veneto (TV), Italy] were divided in groups of 640 larvae per replicate (n=65) for a total of 41˙600 specimens. Each replicate consisted of a plastic box (57x38x16 cm) screened with fine-mesh cotton gauze and covered with a lid provided with 90 ventilation holes (0.05 cm diameter)^14^. Each box was provided with a feeding rate of 448 g once a week. Insects were reared in a climatic chamber at 27±1 °C with 65±5 % of relative humidity^14^. Prepupae, identified by the change in tegument colour, were collected, washed, dried and stored at -80 °C.

**Fish diet production**

BSF full-fat prepupae were freeze-dried and grounded in a mill (Retsch Centrifugal Grinding Mill ZM 1000; Retsch GmbH, Germany) to obtain insect meal for subsequent feed formulation. Two experimental diets were prepared: (i) control diet (Hi0) based on marine (FM and FO) and vegetable protein (pea protein concentrate and wheat gluten meal) resources; (ii) insect-based diet, obtained from the Hi0 formulation including 50% of BSF prepupae meal (Hi50) in substitution of marine resources (both FM and FO). For diet production, all grounded ingredients (0.5 mm) were well mixed and then FO and/or water were added to form a moist blend (Kenwood kMix KMX53 stand Mixer). The blend was pelleted with a grinder (using a 3 mm die meat) and pellets were dried in an oven at 40 °C for 48 hours. Dried pellets were crushed and sieved through a battery of sieves to obtain feed particles ranging from 0.5 to 1.0 mm in diameter. The obtained diets were stored in under vacuum bags and kept at -20 °C until use. Diets ingredient and proximate composition are shown in Supplementary Table S1.

**Supplementary Table S1**. Ingredients (g/kg) and proximate composition (% as feed basis) of experimental diets.

|  | **Hi0** | **Hi50** |
| --- | --- | --- |
| ***Ingredients (g/kg)*** |  |  |
| Fish meal ^1^ | 395 | 198 |
| Pea protein concentrate ^2^ | 120 | 120 |
| BSF meal | - | 230 |
| Wheat gluten meal ^1^ | 130 | 204 |
| Wheat flour ^1^ | 258 | 183 |
| Fish oil ^1^ | 65 | 33 |
| Soy lecitin ^1^ | 8 | 8 |
| Mineral^$^ & Vitamin# supplement | 14 | 14 |
| Binder ^3^ | 10 | 10 |
|  |  |  |
| ***Proximate composition (%)*** |  |  |
| Dry Matter; DM | 94.5 | 93.3 |
| Crude protein; CP | 48.5 | 50.0 |
| Crude lipid; CL | 11.3 | 9.8 |
| Ash | 8.1 | 9.2 |
| Gross Energy (Mj/kg) | 19.9 | 19.2 |

^1^ By Skretting Italia, Mozzecane VR (Italy); ^2^ Lombarda trading srl, Casalbuttano & Uniti (CR, Italy); ^3^ Sodium alginate (Merck KGaA, Darmstadt, Germany). ^$^Mineral supplement composition (% mix): CaHPO_4_*2H_2_O. 78.9; MgO. 2.725 g; KCl. 0.005; NaCl. 17.65; FeCO3. 0.335; ZnSO_4_*H_2_O. 0.197; MnSO_4_*H2O. 0.094; CuSO_4_*5H_2_O. 0.027; Na_2_SeO_3_. 0.067. #Vitamin supplement composition (% mix): thiamine HCL Vit B1. 0.16; riboflavin Vit B2. 0.39; pyridoxine HCL Vit B6, 0.21; cyanocobalamine B12, 0.21; niacin Vit PP, 2.12; calcium pantotenate, 0.63; folic acid, 0.10; biotin Vit H, 1.05; myoinositol, 3.15; stay C Roche, 4.51; tocoferol Vit E, 3.15; menadione Vit K3, 0.24; Vit A (2500UI/kg diet) 0.026; Vit D3 (2400UI/kg diet) 0.05; choline chloride, 83.99.

Feed samples were analysed for dry matter, DM (AOAC #950.46), crude protein, CP (AOAC #976.05) and ash (AOAC #920.153) according to AOAC International^61^. The total lipid fraction (crude lipid, CL) was determined according to the Bligh and Dyer method as previously described in Burja et al. (2007)^62^. The gross energy content (GE) was determined using an adiabatic calorimetric bomb (IKA C7000, Werke GmbH & Co., Staufen, Germany).

**Histology**

Liver, small intestine and spiral valve samples were fixed by immersion in Bouin’s solution for 24 hours, washed three times with 70% ethanol for 15 minutes and finally preserved in a new 70% ethanol solution. After a dehydration through graded ethanol solutions (80, 95 and 100%), samples were washed with xylene (Bio-Optica, Milano, Italy) and embedded in paraffin (Bio-Optica). Solidified paraffin blocks were cut with a microtome (Leica RM2125 RTS, Nussloch, Germany) and 5 µm sections were stained with Mayer haematoxylin and eosin Y (Merck KGaA, Darmstadt, Germany). Sections were observed using a Zeiss Axio Imager.A2 (Oberkochen, Germany) microscope in order to study the hepatic parenchyma and the morphology of small intestine, pyloric caecum and spiral valve. Images were acquired by mean of a combined colour digital camera Axiocam 503 (Zeiss, Oberkochen, Germany). An example of histomorphology of Siberian sturgeon liver and intestine at t_0_ is reported in Supplementary Figure S1.

**
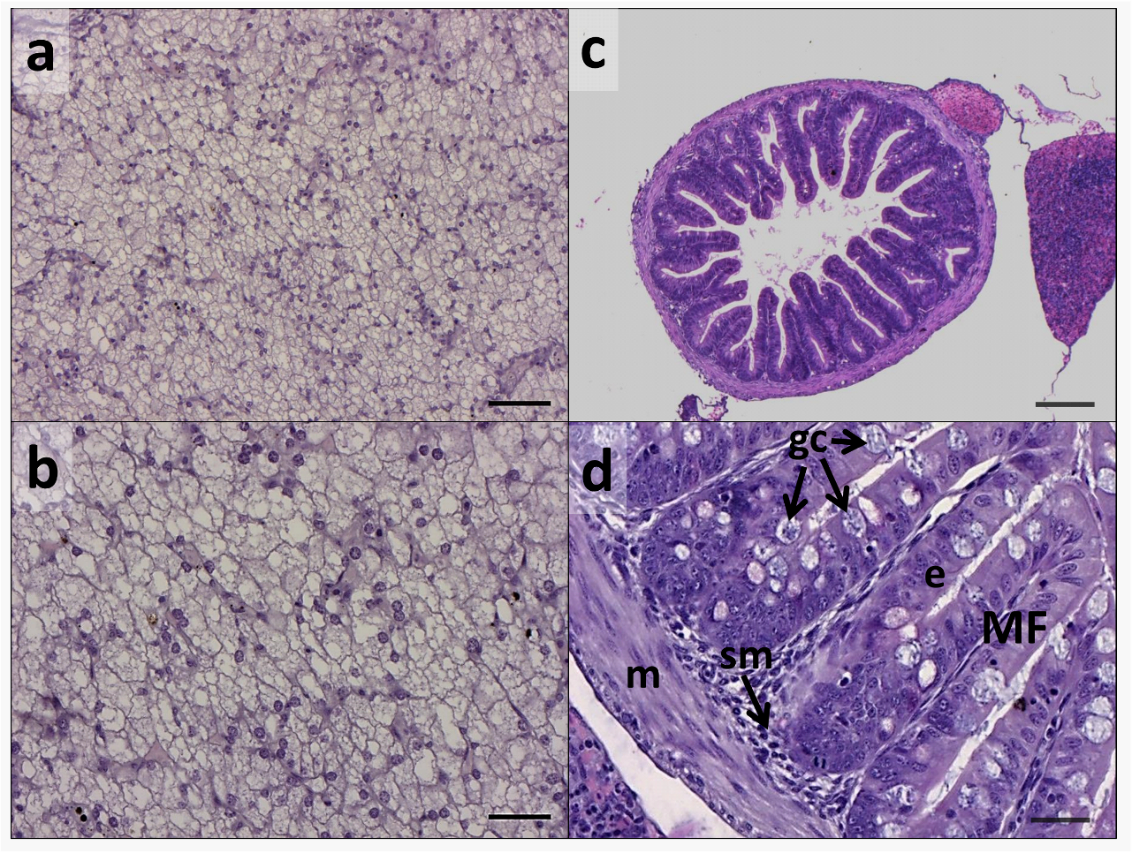
**

**Supplementary Figure S1.** Example of histomorphology of Siberian sturgeon liver and intestine at t_0_. (**a**,**b**) liver; (**c**,**d**) intestine. Scale bars: (**a**) 100 μm; (**b**,**d**) 50 μm; (**c**) 500 μm. Letters: m=muscular layer; sm= submucosal layer; gc=goblet cells; e=enterocytes; MF=mucosal folds.

**FTIR measurements**

From the middle part of each liver and small intestine sample, three thin sections (10 µm thick) were cut at 200 µm intervals by using a chryomicrotome (Microm HM 505 N, Neuss, Germany) and deposited onto CaF_2_ optical windows (1 mm thick, 13 mm diameter) for FTIRM analysis^68^. IR measurements were performed at 20 °C by using a Bruker Invenio FTIR spectrometer equipped with an FPA detector operating at liquid nitrogen temperature (Bruker Optics, Ettlingen, Germany). On each section, two areas were selected on which IR maps were collected in transmission mode in the spectral range 4000-800 cm^-1^ (spectral resolution 4 cm^-1^; spatial resolution 2.56x2.56 µm^2^). IR maps with different size were collected on liver and small intestine sections due to the different morphology and structure of the samples (164x164 µm^2^ size, 4096 pixel/spectra, 128 scans on liver sections, and 328x164 µm^2^, 8192 pixel/spectra, 256 scans on small intestine sections). All raw IR maps were corrected for the contributions of carbon dioxide and water vapor, and then vector normalized to avoid artifacts due to local thickness variations (OPUS 7.0 software, Bruker Optics, Ettlingen, Germany).

**Liver samples***.* False color images representing the topographical distribution of total lipids, fatty acids, unsaturated alkyl chains, proteins and glycogen were built from each IR map. For this purpose, the following spectral Regions Of Interest (ROI) were investigated: 2999-2829 cm^-1^ (total lipids, arbitrary color scale 0-10; LIP); 1775-1724 cm^-1^ (fatty acids, arbitrary color scale 0-3; FA); 3035-2999 cm^-1^ (unsaturated alkyl groups, arbitrary color scale 0-0.5; CH); 1718-1486 cm^-1^ (total proteins, arbitrary color scale 0-10; PRT), and 1073-980 cm^-1^ (glycogen, arbitrary color scale 0-3; GLY). Due to the different absorption of the infrared radiation (molar extinction coefficient, ε) showed by the analyzed macromolecular components, different color scales were adopted; in any case, dark blue color represented the minimum of the infrared absorption, while white/light pink color the maximum one. On all the IR spectra of each map, the integrated areas of the above defined ROI were calculated, and the values used for the following band area ratios: LIP/TBM, FA/TBM, CH/TBM, PRT/TBM, and GLY/TBM. TBM is representative of the total tissue biomass and was calculated by the sum of the integrated areas 2999-2828 and 1801-945 cm^-1^.

**Small intestine samples***.* False color images representing the topographical distribution of total lipids, proteins and carbohydrates were built from each IR map. For this purpose, the following ROI were investigated: 2999-2828 cm^-1^ (total lipids, arbitrary color scale 0-3; LIP); 1718-1486 cm^-1^ (total proteins, arbitrary color scale 0-10; PRT) and 1135-1006 cm^-1^ (total carbohydrates, arbitrary color scale 0-2; CARBO). Due to the different absorption of the infrared radiation (molar extinction coefficient, ε) showed by the analyzed macromolecular components, different color scales were adopted; in any case, dark blue color represented the minimum of the absorption, while white/light pink color the maximum one. From each IR map, ca. 300 IR spectra, representative of the small intestine epithelial absorption portion, were extracted and integrated under the above defined ROI. These values were used for the following band area ratios: LIP/TBM, PRT/TBM, and CARBO/TBM. TBM is representative of the total tissue biomass and was calculated by the sum of the integrated areas 2999-2828 and 1801-945 cm^-1^.

**Molecular analyses**

**RNA extraction and cDNA synthesis.** Total RNA extracted was eluted in 40 µl of RNase-free water (Qiagen) and stored at -80°C until use. The final RNA concentration was determined by a NanoPhotometer P-Class (Implen, München, Germany) and the RNA integrity was verified by GelRed^TM^ staining of 28S and 18S ribosomal RNA bands on 1% agarose gel. The cDNA synthesis was performed with LunaScript RT SuperMix Kit (New England Biolabs, Ipswich, Massachusetts, USA) using 1 µg of total RNA.

**Real-Time PCR.** PCRs were performed in an iQ5 iCycler thermal cycler (Bio-Rad, Hercules, California, USA). According to Vargas-Abúndez et al. (2019)^72^ reactions were set on a 96-well plate by mixing, for each sample, 1µL cDNA diluted 1:10, 5µL of 2x concentrated iQ^TM^ Sybr Green (Bio-Rad, Hercules, California, USA) as fluorescent intercalating agent, 0.3 µM of forward primer and 0.3 µM of reverse primer. The thermal profile for all reactions was 3 min at 95°C and then 45 cycles of 20s at 95°C, 20s at 60°C, and 20s at 72°C. At the end of each cycle florescence was monitored and the melting curve analyses showed in all cases one single peak. Relative quantification of the expression of genes involved in fish growth (insulin-like growth factor 1, *igf1*), stress (heat shock cognate 70-kd protein, tandem duplicate 1, *hsp70.1*) and immune response (tumor necrosis factor a, *tnfa*) was performed. Actin, beta 1 (*actb1*) and glyceraldehyde-3-phosphate dehydrogenase (*gapdh*) were used as internal standards in each sample in order to standardize the results by eliminating variation in mRNA and cDNA quantity and quality. No amplification products were observed in negative controls and no primer-dimer formations were observed in the control templates. The data obtained were analysed using the iQ5 optical system software version 2.0 (Bio-Rad), including GeneEx Macro iQ5 Conversion and Genex Macro iQ5 files. Primer sequences used in this study are reported in Supplementary Table S2.

**Supplementary Table S2**. Primers sequences used in this study

| *Gene* | *Forward primer (5'- 3')* | *Reverse primer (5'- 3')* |
| --- | --- | --- |
| *igf1* | 5'-AGCTGAGCTTGTGGACAC-3' | 5'-AAGCAGCACTCATTCACGAT-3' |
| *hsp70.1* | 5'-ACAGCCATGTTGTATACTGAGTCC-3' | 5'-TGCACACCTTCTCCAGTTCTT-3' |
| *tnfα* | 5'-TGTGTCTGTAGAGCACTCCGAT-3' | 5'-CATGGCCAGCAAGTCGAT-3' |
| *actb1* | 5'-GTTGGTATGGGACAGAAGGACA-3' | 5'-CCAGTTGGTAACAATGCCGT-3' |
| *gapdh* | 5'-CATTTGATGTTGGCTGGGT-3' | 5'-CTTTCTGGGAAGGTGGAGGT-3' |

Gene symbols and gene names are depicted according to the gene zebrafish nomenclature (www.zfin.org).
